# Behavioral Responses to Mammalian Blood Odor and a Blood Odor Component in Four Species of Large Carnivores

**DOI:** 10.1371/journal.pone.0112694

**Published:** 2014-11-10

**Authors:** Sara Nilsson, Johanna Sjöberg, Mats Amundin, Constanze Hartmann, Andrea Buettner, Matthias Laska

**Affiliations:** 1 Department of Physics, Chemistry and Biology, Linköping University, Linköping, Sweden; 2 Kolmården Wildlife Park, Kolmården, Sweden; 3 Department of Chemistry and Pharmacy, Friedrich-Alexander-Universität Erlangen-Nürnberg, Erlangen, Germany; Université Lyon, France

## Abstract

Only little is known about whether single volatile compounds are as efficient in eliciting behavioral responses in animals as the whole complex mixture of a behaviorally relevant odor. Recent studies analysing the composition of volatiles in mammalian blood, an important prey-associated odor stimulus for predators, found the odorant trans-4,5-epoxy-(E)-2-decenal to evoke a typical “metallic, blood-like” odor quality in humans. We therefore assessed the behavior of captive Asian wild dogs *(Cuon alpinus),* African wild dogs *(Lycaon pictus),* South American bush dogs *(Speothos venaticus),* and Siberian tigers *(Panthera tigris altaica)* when presented with wooden logs that were impregnated either with mammalian blood or with the blood odor component trans-4,5-epoxy-(E)-2-decenal, and compared it to their behavior towards a fruity odor (iso-pentyl acetate) and a near-odorless solvent (diethyl phthalate) as control. We found that all four species displayed significantly more interactions with the odorized wooden logs such as sniffing, licking, biting, pawing, and toying, when they were impregnated with the two prey-associated odors compared to the two non-prey-associated odors. Most importantly, no significant differences were found in the number of interactions with the wooden logs impregnated with mammalian blood and the blood odor component in any of the four species. Only one of the four species, the South American bush dogs, displayed a significant decrease in the number of interactions with the odorized logs across the five sessions performed per odor stimulus. Taken together, the results demonstrate that a single blood odor component can be as efficient in eliciting behavioral responses in large carnivores as the odor of real blood, suggesting that trans-4,5-epoxy-(E)-2-decenal may be perceived by predators as a “character impact compound” of mammalian blood odor. Further, the results suggest that odorized wooden logs are a suitable manner of environmental enrichment for captive carnivores.

## Introduction

The hunting behavior of a variety of mammalian predators suggests that they may use the odor of blood to home in on wounded prey [1]–[3]. The behavior of mammalian prey species, in turn, suggests that the odor of conspecific blood may serve as a warning signal and elicits adaptive behavioral responses such as increased vigilance or flight [4]–[8]. The odor of blood may also be an important indicator of the reproductive status in female mammals [9]. Furthermore, mammalian prey species have been observed to display behavioral responses to heterospecific blood odors [10], [11] suggesting that there may be a common olfactory motive in mammalian blood odor that is recognized across species.

Despite the presumed importance of blood odors for both predator/prey relationships and intraspecific chemical communication, surprisingly little is known about the volatiles that comprise the odor of blood in mammals, and even less is known about the constituents of blood odor that elicit behavioral responses in predators and/or prey species. Such knowledge, however, would be useful for both theoretical and practical purposes: it would, for example, allow ethologists to better understand the chemosensory dimension of predator/prey relationships and of intraspecific chemical communication [3]. It would also allow olfactory researchers to further explore the concept of “key compounds” in naturally occurring odor mixtures [12]. Finally, it may allow for the development of more effective chemical repellents, for example along highways or around plantations [13], or more effective environmental enrichment for captive carnivores [14].

Using sophisticated chemo-analytical methods such as gas chromatography-mass spectrometry (GC-MS) and gas chromatography-olfactometry (GC-O), a recent study analyzed the composition of volatiles in mammalian blood and found one substance, trans-4,5-epoxy-(E)-2-decenal, to evoke a typical “metallic, blood-like” odor quality in humans [15]. Humans are extremely sensitive to this odorant and have an extraordinarily low detection threshold of 0.078 ppt –0.33 ppt (parts per trillion) for this aldehyde [16].

It was therefore the aim of the present study (1) to assess behavioral responses of four large carnivore species to mammalian blood odor and to the mammalian blood odor component trans-4,5-epoxy-(E)-2-decenal, (2) to compare their behavioral responses to those towards a fruity odor and a near-odorless control, (3) to compare the behavioral responses between the four species, and (4) to assess the suitability of these odor stimuli as environmental enrichment for captive carnivores.

## Materials and Methods

### Ethics Statement

The experiments reported here comply with the *Guide for the Care and Use of Laboratory Animals* (National Institutes of Health Publication no. 86-23, revised 1985) and also with current Swedish laws. They were performed according to a protocol approved by the ethical board of the Swedish Board of Agriculture (Jordbruksverket, protocol # 31-2647/10).

### Animals

The study was conducted at Kolmården Wildlife Park, near Norrköping, Sweden. The four species studied were:

Asian wild dogs *(Cuon alpinus).* A group of 12 animals, comprising seven females and five males ranging from 1–8 years of age, was observed. The group was composed of one breeding pair plus their offspring. The Asian wild dogs, also known as dholes, were housed in an outdoor enclosure of 2900 m^2^ which contained dens, trees, bushes, and rocks, but mainly consisted of open grassland with a 0.5 m deep water moat along one of its sides.

African wild dogs *(Lycaon pictus).* A group of 12 animals, comprising one female and eleven males ranging from 2–9 years of age, was observed. Nine of the eleven males were offspring of the female. The African wild dogs, also known as painted dogs, were housed in an outdoor enclosure of 5200 m^2^ which consisted of a rocky area in the center with surrounding open grassland areas.

South American bush dogs *(Speothos venaticus).* A group of 10 animals, comprising five females and five males ranging from 1–6 years of age, was observed. The group was composed of one breeding pair plus their offspring. The South American bush dogs were housed in an outdoor enclosure of 1000 m^2^ which contained dens, trees, bushes, and rocks, but mainly consisted of open grassland.

Siberian tigers *(Panthera tigris altaica).* A group of 6 animals, comprising three females and three males ranging from 2–11 years of age, was observed. One of the males was the father of the three females and of one of the males, and he was the brother of the remaining male. The Siberian tigers were housed in an outdoor enclosure of 4800 m^2^ which contained dens, trees, rocks, an area with steep rocky cliffs, and an area with open grassland with both an artificial stream and a water moat along one of its sides.

The outdoor enclosures of all four species were connected to indoor enclosures into which the animals were locked in for cleaning the outdoor enclosures and for removing the odor stimuli after the end of an experimental day.

### Odor stimuli

The four odor stimuli used were:

Blood from a domestic horse *(Equus caballus)*. The rationale for using horse blood was that horse meat is a regular part of the food that all four carnivore species studied here are provided with at Kolmården Wildlife Park. The blood was collected in April 2012 directly after the horse was euthanized and immediately deep-frozen in aliquots of 0.5 ml at –20°C. On the morning of each testing day, five aliquots of horse blood were thawed and warmed up to approximately 25°C.

Trans-4,5-epoxy-(E)-2-decenal (CAS# 134454-31-2). This odorant has been identified as a volatile component in mammalian blood and evokes a typical “metallic, blood-like” odor quality in humans [15]. It was presented at a dilution of 1∶100 (in diethyl phthalate) from a stock solution of 5 mg/ml.

iso-pentyl acetate (CAS# 123-92-2). This odorant has been identified as a volatile component in a variety of fruits and evokes a typical “banana-like” odor quality in humans [17]. It was presented at a dilution of 1∶1,000 (in diethyl phthalate).

Diethyl phthalate (CAS# 84-66-2). This organic solvent is near-odorless and was used both for diluting the two monomolecular odorants mentioned above and as a blank stimulus.

The concentrations mentioned above for the blood odor component and for the fruity odor were chosen in order to provide stimuli that were clearly detectable for humans, but not overwhelmingly strong so that a relatively close contact to the odor source was necessary to detect them.

### Experimental procedure

The four different odor stimuli were presented to the animals using wooden (spruce) logs of 48×7×4.5 cm. Each log was impregnated with 0.5 ml of a given odor stimulus immediately prior to each presentation. The odor stimulus was applied on the two largest surfaces of a log using a micropipette and then spread over the surface using a paint brush. Plastic gloves were used whenever the logs were handled to avoid that they were contaminated with human odor. Five logs, impregnated with the same odor stimulus, were used during a given experimental day.

At the start of each experimental day, five freshly odorized wooden logs were thrown into the outdoor enclosure of a species. Care was taken to present the wooden logs to the animals at positions that differed between experimental days and, at the same time, allowed the experimenter to see all five logs at least at the start of the observation. Immediately after the logs were put in place, the animals were allowed access into the outdoor enclosure and were observed for three hours in the morning and three hours in the afternoon (between 08∶00 a.m. and 16∶00 p.m.). Experiments were only performed on non-rainy days to prevent the odor stimuli from being washed away by the rain. Similarly, experiments were only performed on non-feeding days to prevent the animals from losing interest for the wooden logs due to the presence of food and postprandial inactivity. At least two days were interspersed between consecutive presentations of odorized wooden logs with a given species and care was taken to remove used logs from an enclosure prior to the start of the next presentation. Each of the four odor stimuli was presented to each of the four species for a total of five times in a pseudo-randomized order which resulted in a total of 20 experimental days per species. The experiments were carried out between May and October 2012 (with the South American bush dogs and the Siberian tigers), and between May and October 2013 (with the Asian wild dogs and the African wild dogs).

Continuous sampling was used to record the occurrence of each interaction with a wooden log which was visible to the experimenter. A total of ten behaviors involving different kinds of interaction with or immediate behavioral responses to the inspection of an odorized wooden log were considered (Table 1). During one experimental day per odor stimulus and species, the duration of the behaviors was also recorded using a stopwatch.

**Table 1:** Ethogram of all behaviors considered in the present study.

functional term	description
sniffing	using the nose to investigate a wooden log
licking	using the tongue to investigate a wooden log
biting	using the teeth to investigate a wooden log
pawing	using the paw or claws to scratch a wooden log
toying	moving or otherwise manipulating a wooden log
flehmen	curling of the upper lip and “grimacing” when investigating a wooden log
self-impregnating	rubbing the face or other body part at a wooden log
scent-marking	urinating or defecating or spraying onto a wooden log
orientating	turning head (or ears or eyes) following an interaction with a wooden log
vocalizing	producing sounds during or following an interaction with a wooden log

**Figure 1:**
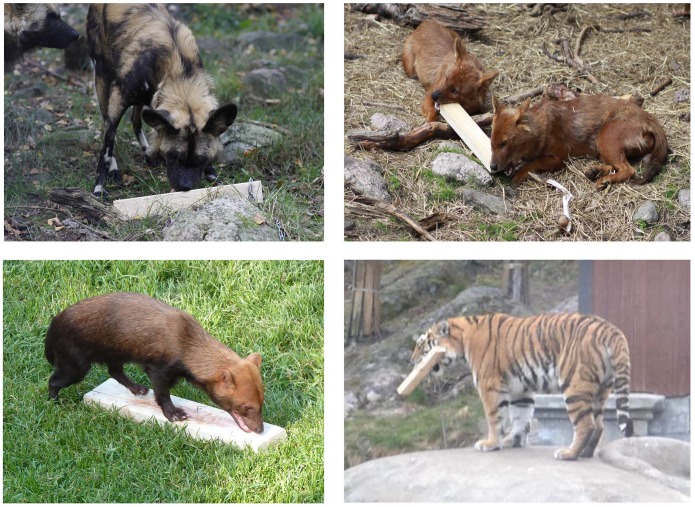
Some of the behaviors considered in the present study. *upper left*: an African wild dog *(Lycaon pictus)* sniffing at an odorized wooden log; *upper right*: two Asian wild dogs *(Cuon alpinus)* biting an odorized wooden log; *lower left*: a South American bush dog *(Speothos venaticus)* performing flehmen on an odorized wooden log; *lower right*: a Siberian tiger *(Panthera tigris altaica)* “toying” with (in this case: carrying) an odorized wooden log.

Figure 1 illustrates some of the behaviors considered in the present study.

### Data analysis

Differences in the frequency of occurrence of behavioral responses between odor stimuli and between species were assessed using the Chi-square test. Between-species comparisons in the duration of behavioral responses were performed using the Mann-Whitney U-test for independent samples. Within-species comparisons of the duration of behavioral responses were performed using the Wilcoxon signed-rank test for related samples. Correlational analyses were performed by calculating Spearman rank-order coefficients r_s_ which were teseted for significance by computing z-scores. As the number of animals differed between species, we calculated the number of interactions per animal by dividing the total number of interactions observed in a given species by the number of animals.

## Results

### Asian wild dogs

Across the 20 experimental days, a total of 458 interactions with the odorized logs was observed. Sniffing was, by far, the most frequently displayed behavior and accounted for 54.6% of all observations, followed by biting (26.0%) and toying (12.4% of all observations). Three of the behaviors, flehmen, scent-marking, and vocalizing were not observed at all (Table 2).

**Table 2:** Number of interactions with the odorized logs in Asian wild dogs (n = 12).

behavior	real blood	blood component	fruity odor	blank control	Total
sniffing	75	110	41	24	250
licking	17	3	0	0	20
biting	51	28	24	16	119
pawing	2	3	1	0	6
toying	13	23	10	11	57
flehmen	0	0	0	0	0
self-impregnating	2	2	0	0	4
scent-marking	0	0	0	0	0
orientating	1	1	0	0	2
vocalizing	0	0	0	0	0
Total	161	170	76	51	458

A comparison between the four odor stimuli showed that the Asian wild dogs displayed a significantly higher number of interactions with the odorized logs when presented with the real blood odor compared to the fruity odor (χ^2^ = 15.02, P<0.0001) and the blank control (χ^2^ = 29.49, P<0.0001). Similarly, the animals displayed a significantly higher number of interactions when presented with the blood odor component compared to the fruity odor (χ^2^ = 17.86, P<0.0001) and the blank control (χ^2^ = 33.39, P<0.0001). There was no significant difference in the number of interactions between the real blood odor and the blood odor component (χ^2^ = 0.07, P = 0.786), and between the fruity odor and the blank control (χ^2^ = 2.10, P = 0.147), respectively.

### African wild dogs

A total of 647 interactions with the odorized logs was observed across the 20 experimental days. Sniffing was, by far, the most frequently displayed behavior and accounted for 64.3% of all observations, followed by licking (16.5%) and biting (12.4% of all observations). Four of the behaviors, flehmen, scent-marking, orientating, and vocalizing were not observed at all (Table 3).

**Table 3:** Number of interactions with the odorized logs in African wild dogs (n = 12).

behavior	real blood	blood component	fruity odor	blank control	Total
sniffing	104	173	85	54	416
licking	10	47	32	18	107
biting	14	44	7	15	80
pawing	1	1	0	0	2
toying	13	8	3	4	28
flehmen	0	0	0	0	0
self-impregnating	0	10	0	4	14
scent-marking	0	0	0	0	0
orientating	0	0	0	0	0
vocalizing	0	0	0	0	0
Total	142	283	127	95	647

A comparison between the four odor stimuli showed that the African wild dogs displayed a significantly higher number of interactions with the odorized logs when presented with the real blood odor compared to the blank control (χ^2^ = 4.31, P = 0.038), but not to the fruity odor (χ^2^ = 0.31, P = 0.575). In contrast, the animals displayed a significantly higher number of interactions when presented with the blood odor component compared to the fruity odor (χ^2^ = 30.01, P<0.0001), the blank control (χ^2^ = 48.78, P<0.0001), and the real blood odor (χ^2^ = 23.37, P<0.0001). There was no significant difference in the number of interactions between the fruity odor and the blank control (χ^2^ = 2.04, P = 0.154).

### South American bush dogs

Across the 20 experimental days, a total of 1582 interactions with the odorized logs was observed. Sniffing was, by far, the most frequently displayed behavior and accounted for 82.5% of all observations, followed by biting (6.6%) and orientating (4.6% of all observations). Two of the behaviors, self-impregnating and vocalizing were not observed at all (Table 4).

**Table 4:** Number of interactions with the odorized logs in South American bush dogs (n = 10).

behavior	real blood	blood component	fruity odor	blank control	Total
sniffing	467	371	261	206	1305
licking	6	2	2	0	10
biting	17	70	9	9	105
pawing	2	8	0	0	10
toying	5	40	6	1	52
flehmen	9	0	1	0	10
self-impregnating	0	0	0	0	0
scent-marking	7	1	8	2	18
orientating	27	29	10	6	72
vocalizing	0	0	0	0	0
Total	540	521	297	224	1582

A comparison between the four odor stimuli showed that the South American bush dogs displayed a significantly higher number of interactions with the odorized logs when presented with the real blood odor compared to the fruity odor (χ^2^ = 35.44, P<0.0001) and the blank control (χ^2^ = 67.41, P<0.0001). Similarly, the animals displayed a significantly higher number of interactions when presented with the blood odor component compared to the fruity odor (χ^2^ = 30.70, P<0.0001) and the blank control (χ^2^ = 60.82, P<0.0001). There was no significant difference in the number of interactions between the real blood odor and the blood odor component (χ^2^ = 0.14, P = 0.712). Finally, the number of interactions with the odorized logs was significantly higher when the animals were presented with the fruity odor compared to the blank control (χ^2^ = 4.86, P = 0.028).

### Siberian tigers

A total of 392 interactions with the odorized logs was observed across the 20 experimental days. Sniffing was, by far, the most frequently displayed behavior and accounted for 49.7% of all observations, followed by biting (14.5%) and toying (11.2% of all observations). Only one of the behaviors, vocalizing, was not observed at all (Table 5).

**Table 5:** Number of interactions with the odorized logs in Siberian tigers (n = 6).

behavior	real blood	blood component	fruity odor	blank control	Total
sniffing	64	93	16	22	195
licking	22	12	1	1	36
biting	31	17	7	2	57
pawing	22	15	4	2	43
toying	21	15	6	2	44
flehmen	1	2	0	0	3
self-impregnating	1	0	0	0	1
scent-marking	1	0	0	0	1
orientating	3	9	0	0	12
vocalizing	0	0	0	0	0
Total	166	163	34	29	392

A comparison between the four odor stimuli showed that the Siberian tigers displayed a significantly higher number of interactions with the odorized logs when presented with the real blood odor compared to the fruity odor (χ^2^ = 47.41, P<0.0001) and the blank control (χ^2^ = 53.31, P<0.0001). Similarly, the animals displayed a significantly higher number of interactions when presented with the blood odor component compared to the fruity odor (χ^2^ = 45.85, P<0.0001) and the blank control (χ^2^ = 51.67, P<0.0001). There was no significant difference in the number of interactions between the real blood odor and the blood odor component (χ^2^ = 0.01, P = 0.969), and between the fruity odor and the blank control (χ^2^ = 0.07, P = 0.789), respectively.

### Comparison between species

The four predator species differed in their total number of interactions with the odorized wooden logs (see Tables 2–5). However, as the number of animals that we studied differed between the four species, we have to consider the number of recorded interactions with an odorized log *per animal* for between-species comparisons.

The South American bush dogs displayed the highest number of interactions with the odorized logs with 158 interactions per animal, and the Asian wild dogs displayed the lowest number of interactions with the odorized wooden logs with only 38 interactions per animal. The Siberian tigers and the African wild dogs fell in between with 65 and 54 interactions with the odorized logs per animal, respectively. Accordingly, the South American bush dogs displayed a significantly higher number of interactions with the odorized logs per animal compared to the three other species (χ^2^≥19.41, P<0.0001 with all three comparisons). In contrast, the African wild dogs, the Asian wild dogs, and the Siberian tigers did not differ significantly in their number of interactions with the odorized logs per animal (χ^2^≤3.09, P>0.05 with all three comparisons).

In all four species sniffing was the most frequently displayed behavior and accounted for at least 49% (Siberian tigers) and up to 82% (South American bush dogs) of all observations. Similarly, in all four species licking, biting, pawing, and toying were among the next most frequently displayed behaviors and together with sniffing these five behaviors accounted for more than 90% of all observations. Finally, vocalizing during or following an interaction with an odorized log was not observed in any of the four species. Accordingly, the rankings from most frequent to least frequent behaviors correlated positively between all four species (Spearman, +0.41≤r_s_≤+0.93 with all six comparisons), and in four of the six cases this correlation was even statistically significant (Asian wild dogs vs. African wild dogs: r_s_ = +0.93, P = 0.0052; Asian wild dogs vs. Siberian Tigers: r_s_ = +0.91, P = 0.0063; African wild dogs vs. Siberian tigers: r_s_ = +0.76, P = 0.0222; South American bush dogs vs. Siberian tigers: r_s_ = +0.77, P = 0.0201).

### Duration of interactions

Table 6 summarizes the average duration of interactions with the odorized logs in the four species. The Siberian tigers displayed significantly longer interactions with the odorized logs compared to the three other species (Mann-Whitney U-test, P<0.05 with all three comparisons). This was true both when considering the four odor stimuli together and when considering the real blood odor, the blood odor component, and the fruity odor separately. In contrast, the Asian wild dogs, African wild dogs, and South American bush dogs did not differ significantly in the duration of their interactions with the odorized logs (Mann-Whitney U-test, P>0.05 with all three comparisons).

**Table 6:** Duration of interactions with the odorized logs (in seconds).

**species**	**real blood**	**blood component**	**fruity odor**	**blank control**	**Total**
Asian wild dogs	4.7±3.0	3.6±2.6	3.9±3.1	4.3±3.6	4.2±2.9
African wild dogs	3.6±1.8	4. 2±3.0	2.7±2.2	4.6±4.3	3.8±3.0
Bush dogs	7.8±7.7	3.2±4.0	3.7±3.3	3.0±2.1	5.6±3.5
Siberian tigers	17.4±7.1	6.2±4.0	7.6±6.6	1.8±0.5	12.6±7.1

given are mean values ± standard deviations.

The South American bush dogs and the Siberian tigers spent a significantly longer time per interaction with the odorized logs when presented with the real blood odor compared to the other three stimuli (Wilcoxon, P<0.05 with all three comparisons per species). The African wild dogs displayed significantly longer interactions with the odorized logs when they were impregnated with the real blood odor or the blood odor component compared to the fruity odor (Wilcoxon, P<0.05 with both comparisons), but not with the blank control (Wilcoxon, P>0.05). The Asian wild dogs did not differ in the duration of interactions with the odorized logs with any of the four stimuli (Wilcoxon, P>0.05 with all six comparisons).

### Variability between sessions

**Figure 2:**
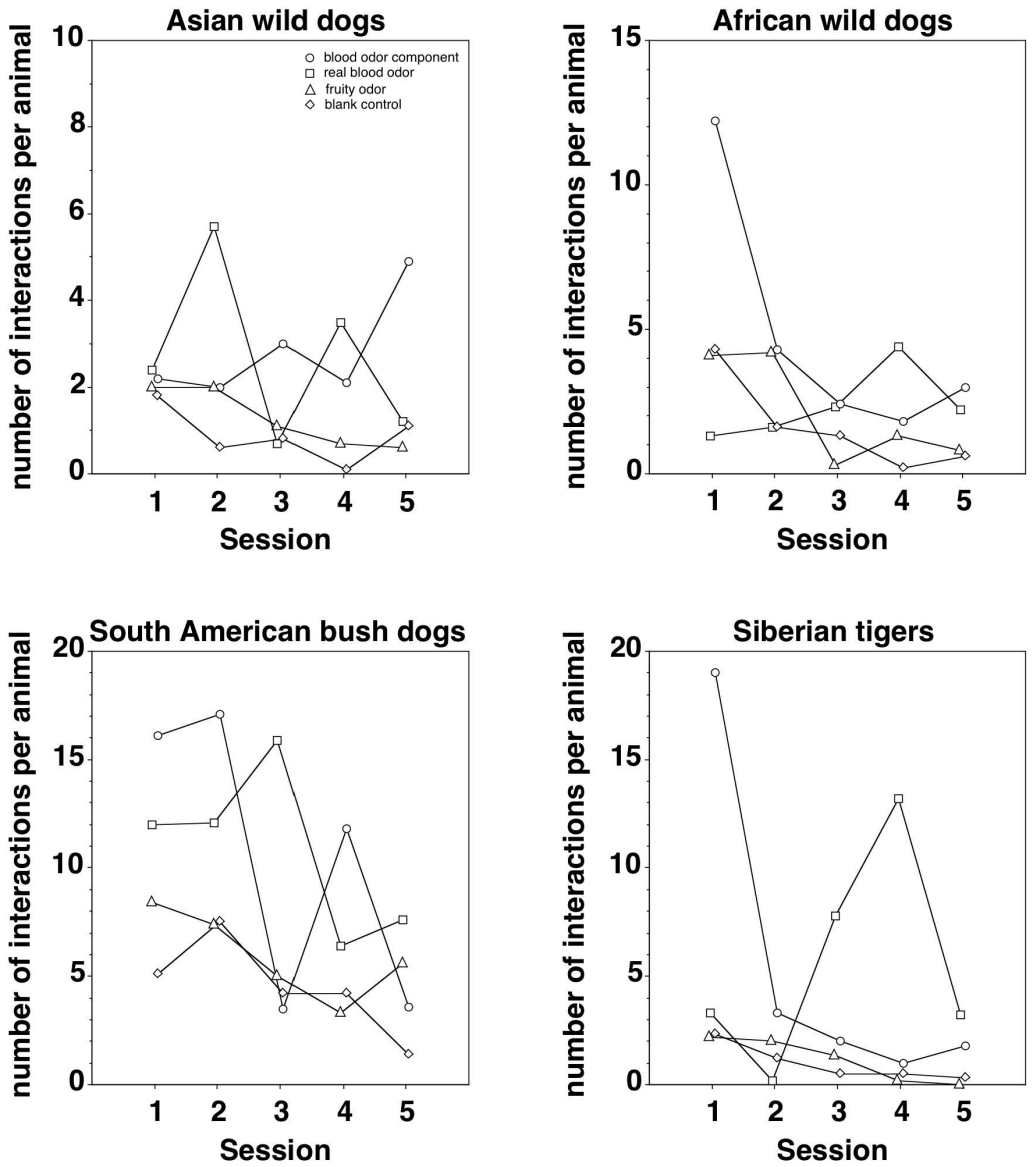
The number of interactions with the odorized logs per animal across the five sessions performed per odor stimulus and species. circles: blood odor component (trans-4,5-epoxy-(E)-2-decenal); squares: real blood odor (horse blood); triangle: fruity odor (iso-pentyl acetate); diamond: blank control (diethyl phthalate).

Figure 2 illustrates the number of interactions with the odorized logs per animal across the five sessions performed per odor stimulus and species. In all four species, and with all four odor stimuli, a considerable session-to-session variability in the number of interactions per animal was observed. Correlational analyses showed a significant decrease in the number of interactions per animal across sessions in the South American bush dogs (Spearman r_s_ = –0.54, P = 0.0193), but not in the Asian wild dogs, African wild dogs, and Siberian tigers (Spearman, –0.44≤r_s_≤–0.24, P>0.05 with all three correlations).

## Discussion

The results of the present study demonstrate that a single blood odor component can be as efficient in eliciting behavioral responses in large carnivores as the odor of real blood. Further, the results show that all four predator species tested here responded differently to prey-associated odors and non-prey-associated odors, respectively. Finally, the results suggest that odorized wooden logs are a suitable manner of environmental enrichment for captive carnivores.

Our finding that trans-4,5-epoxy-(E)-2-decenal was at least as efficient in eliciting behavioral responses in large carnivores as the odor of real blood is not trivial considering that there are hardly any examples so far showing that animals may respond in the same manner to a single food odor component as they do to the real complex odor of a food item. Combined analytical and sensory studies in humans have shown that the complex odor of some, but not all, foods is characterized by so-called “key compounds” (sometimes also referred to as “character impact compounds”) [12]. However, when presented alone, these “key compounds” often resemble, but hardly ever are perceived as being identical with the natural complex odor of the food, thus emphasizing the importance of other components of the mixture as contributors to the odor “gestalt” and identifiability of a food item [18], [19].

In animal studies, nonhuman primates showed no indication at all of associating “key compounds” of natural food items such as acetic esters or monoterpene alcohols found in the odor of tropical fruits with the food items themselves [20]–[22]. Rather, the animals behaved in the same manner as towards other monomolecular odorants that are found in the same fruits, but belong to different chemical classes and are considered not to be “character impact compounds” [23], [24], or to non-food-associated odorants [25].

The same phenomenon that single components of behaviorally relevant odors are usually less effective in animals than the complex odor mixture they are normally part of has been reported repeatedly with predator odors and pheromones: the natural body, faecal or urine odors of predators are usually more effective in eliciting avoidance responses in prey species compared to single components of these odors [26], [27]. Testosterone-dependent volatiles from the urine of male mice that increase aggression among males have been found to be perceptible, but behaviorally ineffective when presented to mice in an aqueous solution and thus outside of a complex urine background odor [28].

**Figure 3:**
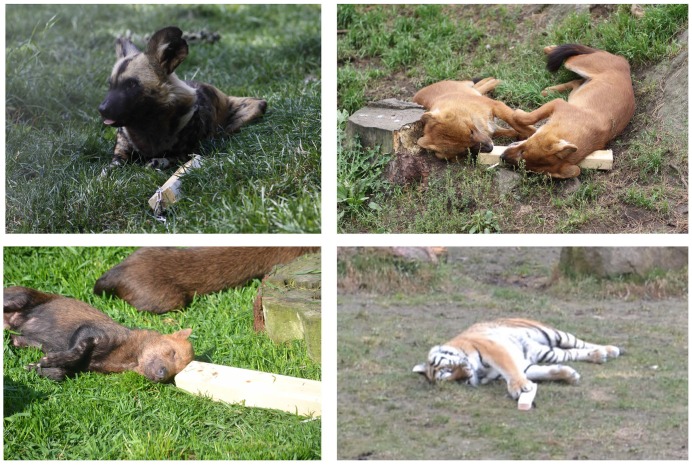
Guarding of or resting close to odorized wooden logs impregnated with either real blood odor or with the blood odor component trans-4,5-epoxy-(E)-2-decenal. *upper left*: an African wild dog *(Lycaon pictus)*; *upper right*: two Asian wild dogs *(Cuon alpinus)*; *lower left*: a South American bush dog *(Speothos venaticus)*; *lower right*: a Siberian tiger *(Panthera tigris altaica).*

Although we cannot be sure that the four predator species tested here indeed associated the odor of trans-4,5-epoxy-(E)-2-decenal with prey or perceived it – similar to humans – as “blood-like”, at the moment this is the most straightforward interpretation of our results. This notion is supported by our finding that three of our four species responded in the same manner to both real horse blood and to the blood odor component – with the fourth species even displaying more interactions with the former compared to the latter stimulus – and differently to the two non-prey-associated odors (the fruity iso-pentyl acetate and the solvent diethyl phthalate). This, too, is not a trivial finding if we consider that mammalian carnivores are known to strongly rely on their sense of smell and therefore generally display a high interest in odor stimuli in their environment [3], [29]. Further, a number of carnivores, particularly members of the canid family, have been reported to regularly self-impregnate with smelly material that is not food-associated [30]–[32]. Additional support for the notion that the four predator species of the present study may indeed have associated the odor of trans-4,5-epoxy-(E)-2-decenal with prey comes from the following observation: all four species tended to guard or to rest close to (or even on top of) odorized wooden logs when they were impregnated with the real blood odor or the blood odor component, but not when they were impregnated with the fruity odor or the blank control (Figure 3). Interestingly, the animals showed a similar behavior on feeding days and tended to guard or rest close to left-overs of their food such as bones.

Future studies are needed to elucidate whether other blood odor components may be equally effective as trans-4,5-epoxy-(E)-2-decenal in eliciting behavioral responses in carnivores or whether this odorant is perhaps indeed the character impact compound of mammalian blood odor, not only in humans [15], [16], but also in nonhuman mammals.

Our finding that the South American bush dogs displayed a significantly higher number of interactions with the odorized logs per animal compared to the other three species raises the question as to possible reasons underlying this difference. Although we cannot rule out the possibility that between-species differences in overall interest in odor stimuli or in behavioral relevance of olfactory information for a given species may account for our data, this seems unlikely because all four species studied here are known to rely on their sense of smell in a variety of behavioral contexts such as hunting and food selection, social communication, mate choice, reproduction, kin recognition, and territoriality [33]–[36]. Rather, it could well be that differences in relative enclosure size might explain the observed between-species difference in the number of interactions with the odorized logs. This notion is supported by the fact that the South American bush dogs had the smallest relative enclosure size with 100 m^2^ per animal and thus they had clearly the highest statistical chance of finding or being close to the odorized logs and thus to interact with them. The Siberian tigers, in contrast, had the largest relative enclosure size with 800 m^2^ per animal. Another possible explanation for our finding that the South American bush dogs displayed significantly more interactions with the odorized logs per animal than the Asian wild dogs, the African wild dogs, and the Siberian tigers is that these species may differ in their overall activity levels in captivity.

We also found that the four predator species differed in the average duration of the interactions with the odorized logs. The Siberian tigers displayed significantly longer interactions with the odorized logs than the other three predator species (see Table 6). Here, too, it seems unlikely that this finding reflects between-species differences in overall interest in odor stimuli or in behavioral relevance of olfactory information for a given species. Rather, these differences may simply reflect the fact that the Siberian tigers displayed the highest proportion of behaviors such as toying and biting (see Tables 1–4) which in all four species lasted longer on average than sniffing, for example.

Finally, the results of the present study suggest that odorized wooden logs are a suitable manner of environmental enrichment for captive predators. This, too, is not a trivial finding if we consider that inactivity or stereotypies are a big problem for zoos keeping large mammalian predators [37].

Environmental enrichment is commonly defined as “an animal husbandry practice that aims to increase species-specific behaviors, increase explorative and interactive behaviors with the environment and reduce abnormal behaviors of captive animals” [38]. A review of olfactory-based enrichment attempts in zoos reported that the presentation of olfactory stimuli can indeed stimulate explorative behaviors and reduce inactivity in a variety of species including large carnivores [14]. However, it was also found that the success of olfactory enrichment may strongly depend on the type of odor used and on the mode of odor presentation and its spatial and temporal distribution. Inappropriate odor stimuli or a continuous odor presentation may be counterproductive and even lead to increased levels of stereotypy and neophobia [39].

The wooden logs used in the present study turned out to be a particularly suitable way of presenting odor stimuli as environmental enrichment for captive predators as they allowed the animals to move the odorized objects and to interact with them in multiple ways (see Figure 1) which would not have been possible if stationary objects such as rocks would have been impregnated with an odor stimulus. Further, the use of wooden logs which can easily be removed from an enclosure (again, as opposed to stationary objects) allowed us to control the temporal distribution of odor presentations and thus to avoid habituation to a given stimulus. In this context it is important to mention that only one of the four species tested here displayed a significant decrease in the number of interactions with the odorized logs across sessions (see Figure 2) and that, in all four species, the number of interactions with the odorized logs did not go down to zero even on the 20^th^ and last test day of testing. None of the four odor stimuli employed here led to an apparent increase in undesirable behaviors such as stereotypies or neophobia, but rather had the desired effect of increasing explorative behaviors and interaction with the environment. We therefore conclude that the presentation of odorized wooden logs is a suitable, cheap and easily applicable manner of environmental enrichment for captive carnivores that may contribute to a sustainable increase in animal welfare, particularly when a larger number of odor stimuli and a pseudorandomized scheme of presentation is employed.
